# Differences in treatment response between migraine with aura and migraine without aura: lessons from clinical practice and RCTs

**DOI:** 10.1186/s10194-019-1046-4

**Published:** 2019-09-06

**Authors:** Jakob Møller Hansen, Andrew Charles

**Affiliations:** 1 Danish Headache Centre and Department of Neurology, Rigshospitalet Glostrup, Valdemar Hansen Vej 5, DK-2600 Glostrup, Denmark; 2 UCLA Goldberg Migraine Program, Department of Neurology, University of California Los Angeles, Los Angeles, CA USA

**Keywords:** Migraine, Migraine aura, Migraine treatment, Clinical trials, Review

## Abstract

Migraine is a major public health problem afflicting approximately 10% of the general population and is a leading cause of disability worldwide, yet our understanding of the basis mechanisms of migraine remains incomplete. About a third of migraine patients have attacks with aura, consisting of transient neurological symptoms that precede or accompany headache, or occur without headache. For patients, aura symptoms are alarming and may be transiently disabling. For clinicians and scientists, aura represents an intriguing neurophysiological event that may provide important insight into basic mechanisms of migraine. Several observations point toward important differences between migraine with and without aura. Compared with migraine without aura, migraine with aura has different heritability, greater association with different conditions including stroke, different alterations of brain structure and function as revealed by imaging studies. A number of studies also indicate that migraine with aura may respond differently to acute and preventive therapies as compared to migraine without aura. The purpose of this review is to provide an overview of these differences in treatment responses, and to discuss the possibility of different therapeutic strategies for migraine with vs. without aura.

## Background

Migraine is the most prevalent neurological disease [1] afflicting a large part of the population across the world [2] and ranks the 2nd leading cause of years lived with disability [3] especially in the young and middle-aged [4].

Migraine has been vividly depicted since the dawn of medicine with the first accounts of attacks of migraine with aura dating back more than two millennia [5]. Still today, migraine aura is both an alarming symptom to patients and an intriguing phenomenon to clinicians and scientists.

Several observations point toward important differences between migraine with and without aura. Migraine with and without aura show distinct familial occurrence and mode of inheritance, suggesting different etiology [6]. Migraine with aura is associated with an increased risk of ischemic stroke, whereas no increased risk is associated with migraine without aura [7, 8]. A number of other disorders are also associated with migraine with aura, but not with migraine without aura [9].

Imaging studies suggest that structural brain changes are more prevalent in those with migraine than in controls, and some of these changes are most pronounced in migraine with aura [10]. During attacks, cerebral blood flow changes may differ between migraine with and without aura [11, 12].

Whether migraine with aura represents a distinct disorder or is simply a part of the spectrum of migraine remains an open question. Regardless of the answer to this question, however, there may be differences in therapeutic responses of individual attacks to acute therapies, and also in the efficacy of preventive approaches for migraine with vs. without aura.

### What is migraine aura?

Up to 1/3 of migraine patients experiences aura [13]; reversible transient focal neurological symptoms arising from the cortex or brainstem [14]. The diagnostic criteria for migraine with aura are listed in Table 1.

**Table 1:** ICHD-3 criteria for migraine with aura [14]

A. At least two attacks fulfilling criteria B and C	
B. One or more of the following fully reversible aura symptoms:	
a. visual	
b. sensory	
c. speech and/or language	
d. motor	
e. brainstem	
f. retinal	
C. At least two of the following four characteristics:	
a. at least one aura symptom spreads gradually over ≥5 min, and/or two or more symptoms occur in succession	
b. each individual aura symptom lasts 5–60 min1	
c. at least one aura symptom is unilateral2	
d. the aura is accompanied, or followed within 60 min, by headache	
D. Not better accounted for by another ICHD-3 diagnosis.	
1 When for example three symptoms occur during an aura, the acceptable maximal duration is 3 × 60 min. Motor symptoms may last up to 72 h	
2 Aphasia is always regarded as a unilateral symptom; dysarthria may or may not be	

Among patients with migraine with aura, 99% of patients report visual symptoms in at least some of their attacks [15], but symptoms may also include sensory, speech/language and motor symptoms and sometimes also higher cortical functions.

Clinical observations suggest a high degree of clinical variability in migraine aura both between patients [16] and from one attack to the next [17]. Most patients with migraine with aura also have migraine attacks without aura. In patients with attacks with and without aura, trigger factors are reported more often for attacks without aura [18].

Aura typically begins before the headache, but in a significant number of patients, headache and aura may occur simultaneously [19] and aura may even occur in the absence of headache [20, 21].

**Fig. 1:**
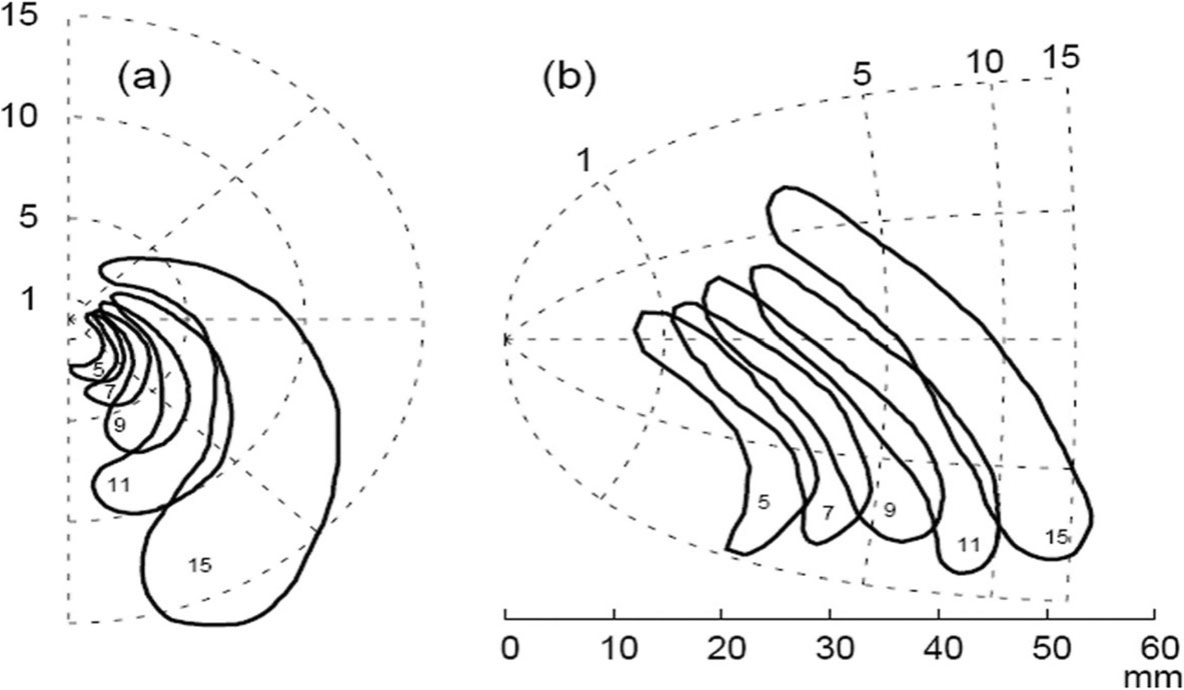
Typical propagation pattern of a visual migraine aura. The figure depicts the right visual hemifield and the travelling visual migraine aura, with the numbers indicating the time passed (in minutes) since first occurrence (**a**). Here, the visual disturbance is projected onto a flat model of the primary visual cortex by reversed retinotopic mapping (**b**). Used with permission and adapted from [23]

Lashley provided the first quantitative recording of the temporal spread of the migrainous scotomas and fortification spectra [22]. The aura is often perceived as having a “spreading” character (Fig. 1), and aura symptoms normally occur in succession suggesting an underlying mechanism that propagates slowly through adjacent brain tissue.

The temporal and spatial characteristics of the spread of the migraine visual aura are similar to those that would be expected to be produced by cortical spreading depression (CSD) discovered by Leão [24]. CSD is a wave of depolarization of neuronal and glial membranes that propagates in brain tissue at a rate of approximately 3 mm/minute fitting with the clinical symptoms and therefore considered a likely mechanisms of the migraine visual aura [25].

### Potential differences in disease mechanisms in migraine with and without aura

#### Cortical spreading depression

The migraine aura served to differ between migraines two major clinical forms (Table 1). The exact link between aura and headache is target for scientific scrutiny; if aura causes headache, treating aura will ease the pain. Preclinical studies have suggested CSD leads to headache [26]. CSD may cause inflammation and the release of nociceptive substances, vasodilation and activation of nociceptive afferents [27]. Animal studies found that CSD was associated with an increased permeability of the blood–brain barrier [28].

In humans, however, aura may not always precede headache [19] and the blood-brain barrier remained intact during the headache phase of spontaneous migraine with aura [29] and attacks without aura [30] as well as during GTN-induced migraine [31].

Most migraine patients do not have auras, and auras may also happen without ensuing headache.

The fact from clinical trials of tonabasat [32] that auras may be reduced without reducing migraine headache without aura speak against subclinical CSD happening silently in attacks without aura.

#### Imaging

Two metaanalyses have looked at the relationship between migraine and white matter abnormalities on MRI. Based on seven studies, it was found that patients with migraine has an four times increased risk for WMA, OR 3.9 (95% CI 2.3–6.7) [33].

An updated meta-analysis was based on six population-based and 13 clinic-based studies [10]. In general, structural brain changes were more prevalent in migraineurs than in controls. Compared to controls, the risk was higher in migraine with aura, OR 1.68 (95% CI 1.07–2.65), but not in migraine without aura OR 1.34; 95% CI 0.96–1.87), without any difference of WMAs in patients with and without aura.

The risk for infarct-like lesions did not differ between migraine with aura and controls, or between migraine without aura and controls. The risk in migraine with aura was, however, greater than in migraine without aura OR 1.44 (95% CI 1.02–2.03), based on just two studies [34, 35].

Structural neuroimaging is being used to search for imaging biomarkers that may potentially be used in decisions regarding migraine diagnoses, treatment and prognostication. In patients with migraine with aura different pattern of cortical thickness was observed in various cortical regions [36]. Biomarkers may also exist outside the brain, as it was reported that migraine with, but not without, aura was associated with foveal and peripapillary vascular decrements on optical coherence tomography angiography [37].

#### Migraine, aura and cerebrovascular disease

The available data of the association between migraine and ischemic stroke have been examined in four pooled meta-analyses [9, 38–40]. Migraine as such was found to be associated with an increased risk of stroke, but when results were stratified according to aura or no-aura, is was consistently found that aura is associated with a 2-fold increase in the risk of ischemic stroke.

#### Endothelial dysfunction

Endothelial dysfunction might play a role connecting migraine and stroke. Endothelial dysfunction in the broadest sense would lead to a procoagulatory [41], proinflammatory and proliferative state, and ultimately; artherosclerosis. A clinic-based study tested younger women for changes in coagulation, inflammation and oxidative stress [42]. In total 125 migraine patients were included, evenly divided between migraine with and without aura. The authors reported a robust association between a number of biomarkers of endothelial activation and migraine, especially for migraine with aura.

#### Vasoconstriction and the use of anti-migraine-drugs

Some of the anti-migraine medications as ergots and triptans have vasoactive actions. In several studies, triptan treatment is not associated with increased risk of stroke, even in the setting of overuse. In two population-based studies, there was no evidence that triptans lead an increased risk of vascular events [43]. Ergot alkaloids in migraine patients has not been statistically verified as a stroke risk factor but high ergotamine consumption is possibly associated with an increased risk of serious ischemic complications [44]. For themost used anti-migraine drug, triptans, the current best evidence does not suggest any increase in cerebrovascular risk, and if one exist, it must be fairly limited [45].

#### Patent foramen ovale, migraine and aura

Patent foramen ovale (PFO) is a common congenital cardiac defect that may serve as right-to-left shunt for paradoxical embolism and cause ischemic stroke, especially in the young [46]. In patients with cryptogenic stroke with concomitant migraine, there is a high prevalence of PFO (79%), and in the group with migraine with frequent auras an astounding 93% had PFO [47].

A systematic review [48] demonstrates that compared to the general population, migraine is associated with a higher prevalence of patent foramen ovale, especially for migraine with aura.

As for tonbasat, PFO closure does not seem to benefit migraine patients in general [49, 50], but the patients with aura show better results and aura may be reduced, although new studies are needed to verify this. These results do, provide a link between clinical observation and animal studies showing that microemboli can cause CSD [51] supporting a causative role for right-to-left shunt in migraine aura.

Interestingly, one study retrospectively examined the effect intensified anticoagulant regimen after PFO closure, and found that the combination of clopidogrel and aspirin resulted in fewer patients (12.2%) reported aura or migraine with aura or aura compared to aspirin alone (42.3%) [52].

In a state-of the-art RCT of transcatheter ASD closure, the use of clopidogrel and aspirin, compared with aspirin alone, resulted in a lower monthly frequency of migraine attacks over 3 months, but no difference between the two groups regarding the type of migraine (aura vs no aura) [53].

#### Psychiatric and cognitive symptoms associated with migraine with aura

In a large population-based study, depression and depression with comorbid anxiety disorder were more likely in women with MA than in MO, with an OR around 1.7 [54]. No difference was found in men, and the exact importance of this result needs validation.

Some, reversible, cognitive impairment may be reported during migraine attacks [55]. A study evaluated cognitive functions and psychological symptoms in MO and MA, and although migraine patients in some aspects differed from controls (lower scores in delayed memory and set-shifting performances), no clear differences emerged between MA and MO [56].

### Treatment of migraine with aura

Current guidelines recommend that the same treatment be used in migraine with and without aura.

This is not surprising because almost all studies of both acute and preventative migraine treatments are based on mixed populations of patients that include those with migraine with aura and/or without aura, and the treatment effect based on this diagnosis or attack subtype is rarely reported.

### Targeting the migraine aura

The aura is transient in nature, and acute treatment targeting the aura itself should have an immediate effect to yield meaningful clinical efficacy (although, as discussed below, there may also be differences in acute therapies with regard to headache associated with aura within an attack). Similarly, preventive treatment may be given in an attempt to reduce the aura frequency, but the goal is typically to reduce the frequency of attacks in general.

There are no currently available treatments that are well-proven treatment to abort or shorten the aura symptoms, but a number of treatments have been tested, often in case series or un-blinded studies.

Glutamate receptors inhibitors, particularly NMDA receptor antagonists, have been reported to inhibit the initiation and propagation of CSD, indicating that activation of NMDA receptors play a key role in generating CSD [57]. Ketamine is an NMDA receptor antagonists, that was tested in prolonged aura in 11 patients with hemiplegic migraine, 5 of whom reported shorter aura duration [58]. In a double-blinded, randomized parallel-group controlled study, the effect of 25 mg intranasal ketamine was compared to 2 mg intranasal midazolam as an active control. In the 18 subjects with migraine with prolonged aura completing the study, ketamine reduced the severity but not duration of aura, while midazolam was ineffective [59]. The usefulness of ketamine for aura-treatment in classical migraine aura remains to be established.

Based on clinical experience and the assumption that migraine is associated with defective platelets [60], aspirin have been tested for migraine aura prophylaxis. In an observational case series of 49 patients with migraine given aspirin 80 mg daily, the aura frequency was reduced in 39 of the 42 cases (93%) and the complete cessation of auras in 20 (48%) [61].

Another, retrospective study of 203 patients with migraine with aura, out of whom 95 (46.8%) used acetylsalicylic acid and reported a “positive effect” and a significant reduction in aura duration (from 36 to 22 min) [62]. Despite being readily available and well-tolerated, the use of daily aspirin prophylaxis in patients with migraine aura should be studied further, preferably in a larger double-blind, placebo-controlled study.

A small open-label trial of levetiracetam included 16 patients with migraine with aura, and led to reduction in monthly attacks, and complete disappearance of aura in 43% (7/16) and a reduced aura duration in the remaining patients [63]. Another potential way of targeting CSD is using amiloride a blocker of epithelial sodium channels based upon the role of the acid-sensing ion channel 1 in CSD in animal studies. In a small open-labelled pilot study, amiloride reduced aura and headache symptoms in 4 of 7 patients with otherwise intractable aura [64]. Finally, the effect of ginkgolide B, a herbal constituent extract from *Ginkgo biloba* tree leaves has been tested for the prophylactic treatment of migraine with aura (MA). In open-label study in 50 women suffering from migraine with typical aura, or migraine aura without headache the compound lead to a reduced number of attacks and aura duration [65], a finding confirmed in a subsequent open-label study [66].

### Acute treatment of migraine headache in migraine with aura

#### Triptans

Previous studies indicated that triptans, specifically sumatriptan, eletriptan, and zolmitriptan [67–69], were not effective in relieving migraine when taken during the aura phase of an attack. Sumatriptan or placebo injected at the onset of aura resulted in a similar number of patients with moderate or severe headache. The aura duration was 25 min in the treatment group vs 30 min in the placebo, neither statistically not clinically significant [67]. The eletriptan study found no significant difference in the proportion of patients not developing moderate-to-severe headache within 6 h post-dose of eletriptan (61%, 22/36 patients) versus placebo (46%, 19/41); *P* = 0.25). Despite the high placebo responder rate, these figures translated to a therapeutic gain of 15% and a NNT of 6.7 for eletriptan taken during aura.

In a small, four-way crossover open-label study, treatment with sumatriptan 100 mg during aura prevented the development of the headache in 34 out of 38 attacks (89%), and other studies in selected and complicated patients, suggest that triptans may reduce headache when taken during the aura phase [70–72].

In the seminal RCT of subcutaneous sumatriptan [73] the primary endpoint was pain relief at one hour. Patients who had aura with their migraine and those without aura responded similarly to sumatriptan with a therapeutic gain of 43% for attacks with aura and 49% for attacks without aura. Although the difference in response was not statistically significant, it was consistent with our report of numerically better treatment effect of sumatriptan in attacks without aura (see below).

In another of the first RCTs of sumatriptan (200 mg vs placebo), patients were asked to treat three attacks, as soon as they were aware of a migraine with aura. Sumatriptan reduced the severity of the first migraine attack (sumatriptan 63% vs. 33% placebo), but the severity of the next two attacks did not differ, likely due to a higher placebo respons [74].

Systematic reviews of sumatriptan trials found insufficient data to carry out any sensitivity analyses for participants with and without aura [75–77].

We have therefore previously conducted an analyses on data from the largest available database of acute treatment response - the sumatriptan/naratriptan aggregate patient (SNAP) database [78] to perform a post-hoc comparison of the efficacy of acute treatment in individual migraine attacks with aura vs. without aura, and for sumatriptan we also compared patients with a diagnosis of MA to MO [79]. The pooled pain free rates 2 h post-dose for sumatriptan 100 mg were significantly higher in patients treating attacks without aura (32%), compared to the group who treated attacks with aura (24%),(*P* < 0.001). The relative risk for pain freedom 2 h post-dose for attacks without aura was 1.33 (95% CI: 1.16–1.54). The NNT for 2 h pain free was 4.4 for attacks without aura and 6.2 for attacks with aura. Although the absolute difference in treatment between attacks with and without aura is small, an 8% overall difference in efficacy based on the type of attack has the potential to have a significant impact on the outcome of a clinical trial.

This post-hoc analysis of pooled data from multiple randomized trials indicates that sumatriptan is less effective as acute therapy for migraine attacks with aura compared to attacks without aura. Different responses of migraine with vs. without aura to acute therapies may provide insight into underlying migraine mechanisms and influence the choice of acute therapies for different types of migraine attacks.

#### Transcranial magnetic simulation and other treatments

Transcranial magnetic simulation (TMS) is a non-invasive procedure designed for the acute treatment of migraine with aura, based on the principle that a single pulse of transcranial magnetic stimulation interrupt the wave of CSD during a migraine aura. In a randomized, sham-controlled trial including 164 patients, the 2-h pain-free response rates were 39% in the active group vs 22% in the sham group, giving a therapeutic gain of 17% and a NNT of 5.9 [80]. Another study of sTMS, included patients with aura (*n* = 10) and without aura (*n* = 25) reported an overall decrease in pain score of 75% from baseline after treatment with TMS, and in individuals with an aura (n = 10), relief was 100% and immediate [81].

Another study including both patients with (*n* = 13) and without aura (*n* = 14) reported no difference between sTMS and sham for migraine attacks or migraine days during 8 week trial, but did not evaluate aura separately [82].

A recent systematic review based on 5 studies concluded that sTMS may be effective for migraine with aura, but found no effect of sTMS in chronic migraine [83]. Blinding is an issue in all these trials, but the method is safe and represents an alternative to systemic therapies. Replication of the results are warranted, and the acute effect on aura has not been described.

In a small but randomized, placebo-controlled, double blind study of dipyrone (Metamizol) in migraine with and without aura, the authors reported more pronounced effect on intensity of pain, nausea, photophobia, and phonophobia in patients without aura than patients with aura after placebo administration [84].

One study examined the intensity of pain and associated symptoms after placebo administration in patients with migraine with aura and migraine without aura. After placebo administration, reduction of symptom intensity (pain, nausea, photophobia, and phonophobia) in patients with migraine without aura was significantly greater than that observed in patients with migraine with aura, and the authors suggest that future studies should stratify patients according to the presence versus absence of aura [85]. If the placebo rate differs between migraine with and without aura, studies reporting therapeutic gains and NNT might be skewed.

Magnesium is an important intracellular mediator and low cortical magnesium levels may increase NMDA receptor sensitivity leading to glutamate-induced CSD [86]. The effect of magnesium was tested in patients with migraine without aura and migraine with aura in a randomized, double-blind, placebo-controlled study. In MO-patients there was no statistically significant difference in the patients who received magnesium sulphate vs. placebo in pain relief (TG 17%, NNT 6 at 1 h). In MA-patients, however, a statistically significant improvement of pain and of all associated symptoms was reported compared with controls (TG 36.7%, NNT 2.7 at 1 h) [87]. A recent retrospective study confirmed that magnesium infusion improved pain score, but found no difference between attacks with and without aura [88]. These findings await replication in larger studies.

### Preventive therapy

Preventive treatment for migraine with aura has historically been for the most part similar to treatment for migraine without aura, and most studies examining preventive migraine treatment have been done in mixed populations of MA and MO.

A systematic review with meta-analysis of preventive pharmacologic migraine treatments found that no trials directly compared drug effects in patients with and without aura [89].

In animal studies chronic treatment with a number of widely prescribed migraine prophylactic drugs (topiramate, valproate, propranolol, amitriptyline, and methysergide) suppressed CSD by 40 to 80%, suggesting that CSD in rodents is a translational model for migraine prophylaxis [90]. In rats lamotrigine and valproate also suppressed CSD [91]. If CSD plays a primary role in headache, it might be expected that patients with migraine with aura are more likely to respond to prophylaxis with CSD -suppressing drugs than patients without aura. This, however, has never been shown in any systematic way.

OnabotulinumtoxinA is approved for the treatment of *chronic* migraine. In patients using OnabotulinumtoxinA for preventive migraine treatment, some authors found that aura predict a more favorable outcome [92], whereas others did not [93].

#### Glutamatergic targets for migraine with aura

The link between glutamate and migraine includes increased levels of glutamate in migraine patients, genetics suggesting aberrant glutamate signaling in migraine, and in-vivo evidence of glutamate in pain transmission, central sensitization, and cortical spreading depression [57].

Memantine is anantagonist at glutamatergic NMDA receptors, and in a randomized, double-blind, placebo-controlled trial in patients with migraine without aura, memantine led to an significant reduction in headache [94].

Based on the proposed mode of action, memantine should also work in migraine with aura. In a prospective, open-label trial of 127 patients, 81 patients 74.3% had at least one episode of migraine with aura during baseline, Memantine was effective, but detailed data on attacks with and without aura are not presented [95]. Another retrospective study also found mementine effective in both migraine with and without aura. Out of 20 patients with migraine with aura, 16 reported that it reduced the frequency of aura as well as headache [96].

Lamotrigine, blocks voltage-sensitive sodium channels and may also suppress the release of glutamate in the CNS. CSD is associated with the release of glutamate into the extracellular space and lamotrigine has been shown to suppress CSD in the rat brain [91].

Based on a positive pilot-study [97], a larger open-label study of lamotrigine examined the prevention of migraine aura and reported that more than 75% of patients reported a reduction in aura frequency of more than 50%.Also, more than three quarters of those patients with a reduction of aura symptoms experienced a significant reduction of frequency of migraine attacks. The authors suggested a potential role of aura-like events and possibly cortical spreading depression as a trigger for trigeminal vascular activation, and subsequently the development of migraine headaches [98].

Another open-label study examined whether lamotrigine could cause a > 50% reduction in the mean frequency of migraine auras. Response was considered as excellent (> 75% reduction) in 21 cases (70% of responders). Auras reappeared in 9 out of 13 patients when lamotrigine was stopped, but could be controlled as soon as the drug was reintroduced [99].

Tonabersat is benzopyran derivative that blocks the cortical spreading depression. The drug was tested in two dose-ranging, double-blind, randomized, placebo-controlled, parallel-group trials, and even though more patients given tonabersat than given placebo experienced relief of headache pain, the study did demonstrate any significant effect [100]. In a separate RCT, focused on migraine with aura, tonabasat was found to prevent attacks of migraine with aura but not those without aura [32], suggesting that tonabasat could be a selective drug for migraine with aura.

Topiramate has been shown to inhibit cortical spreading depression and nitroglycerin-evoked hyperalgesia in animal models. Topiramate has multiple potential mechanisms of action and modulates trigeminovascular transmission within the trigeminothalamic pathway, potentially by interaction with the glutaminergic kainate receptor [101]. In the Prolonged Migraine Prevention study with Topiramate (PROMPT) [102], post-hoc analysis showed a similar percentage reduction in MA compared with MO patients (43% vs. 44% reduction in number of migraines). The authors also state that the reduction in auras during topiramate treatment tended to be somewhat more pronounced than the reduction in migraine headaches [103].

Another study randomized 213 subjects from 27 centers to topiramate or placebo [104]. Seventy-five (35.5%) subjects in the ITT population had migraine with aura. The change in mean monthly migraine frequency was not different between topiramate and placebo. In a subgroup analysis, a significant difference was found in MA patients between topiramate (*n* = 46) and placebo (*n* = 29). In pediatric patients, the presence of “visual symptoms” was not a predictor for treatment response to topiramate [105].

#### Transcranial magnetic simulation

Transcranial magnetic simulation (TMS) is a non-invasive procedure designed for the acute treatment of migraine with aura, based on the principle that a single pulse of transcranial magnetic stimulation interrupt the wave of CSD during a migraine aura. In a randomized, sham-controlled trial including 164 patients, the 2-h pain-free response rates were 39% in the active group vs 22% in the sham group, giving a therapeutic gain of 17% and a NNT of 5.9 [80]. Another study of sTMS, included patients with aura (*n* = 10) and without aura (*n* = 25) reported an overall decrease in pain score of 75% from baseline after treatment with TMS, and in individuals with an aura (n = 10), relief was 100% and immediate [81].

Another study including both patients with (*n* = 13) and without aura (*n* = 14) reported no difference between sTMS and sham for migraine attacks or migraine days during 8 week trial, but did not evaluate aura separately [82].

A recent systematic review based on 5 studies concluded that sTMS may be effective for migraine with aura, but found no effect of sTMS in chronic migraine [83]. Blinding is an issue in all these trials, but the method is safe and represents an alternative to systemic therapies. Replication of the results are warranted, and the acute effect on aura has not been described.

### Implications for future trials

Patients with migraine with aura, may have attacks with and without aura and most had more than one subtype of migraine with aura [106]. Even though few patients report only MA, the group having both MO and MA is often substantial, and is it therefore important to classify each individual attack being treated according to the International Classification of Headache Disorders, as suggested by the International Headache Society clinical trials subcommittee [107]. The trials of tonerbasat showed efficacy in migraine with aura, but not in migraine without aura, suggesting that migraine with and without aura should be studied separately [108].

Trials of migraine prophylactic drugs generally focus on reduction in the number of migraine days as the key efficacy parameter, and little attention has been paid to the influence of these drugs on the occurrence of auras.

Future studies should have a clear distinction between aura and non-aura headaches. Other modifying factors, as attack frequency and treatment (acute and prophylactic) is also needed to understand how imaging changes is related to clinical outcomes.

## Conclusion

The present findings indicate that some treatments may have different efficacy in attacks of migraine with aura vs. without aura. It is an important confounder that many patients diagnosed with migraine with aura occasionally have attacks of migraine without aura – and vice versa.

It is unresolved whether patients should employ different treatment strategies based on a previous history of migraine with versus without aura, or based on the presence or absence of aura during an individual attack. Understanding these differential responses to therapy may be an important step to personalized medicine in acute migraine treatment.

## Data Availability

The litterature and datasets used for the current study are available from the corresponding author on reasonable request.
